# Complete genome sequences of two dye-decolorizing aeromonads isolated from a stormwater drain in Hong Kong

**DOI:** 10.1128/mra.00524-25

**Published:** 2025-07-31

**Authors:** P. S. K. Leung, K. M. Leung, G. K. K. Lai, S. D. J. Griffin

**Affiliations:** 1 Shuyuan Molecular Biology Laboratory, The Independent Schools Foundation Academy 257202 , Hong Kong, China; University of Southern California, Los Angeles, California, USA

**Keywords:** *Aeromonas hydrophila*, *Aeromonas dhakensis*, dye-degradation, bioremediation

## Abstract

*Aeromonas hydrophila* subsp. *hydrophila* PSKL.DP and *Aeromonas dhakensis* PSKL.DM were isolated from a stormwater drain in Hong Kong. Their complete genomes—single chromosomes of 4,844,288 bp (61.50% G+C) and 4,820,799 bp (61.49% G+C), respectively—were established through hybrid assembly.

## ANNOUNCEMENT

*Aeromonas* species comprise Gram-negative facultative anaerobes found as non-spore forming, flagellated rods, that are ubiquitous in freshwater and brackish environments (1). They have been isolated from drinking water, soil, raw meats, and agricultural produce (2–4); from the gastrointestinal tracts of aquatic animals, including birds (5–7); and even from microplastics (8). While *Aeromonas* spp. may be linked to cases of human infection, likely through untreated water (2), their versatile biochemistry has been applied to the bioremediation of triarylmethane dyes (9) and heavy metals (10). They are also useful as producers of industrial enzymes and polyhydroxyalkanoate (11).

Here, two aeromonads were isolated from a stormwater drain in Hong Kong (22.264420°N, 114.130481°E; 2022-06-02). A 1 mL sample of rainwater from a collector drain was serial diluted to 0.1% v:v in 0.9% w:v saline before spreading a 100 µL aliquot onto CHROMagar Vibrio plates and incubating for 48 h at 27°C (this temperature was used throughout). PDKL.DP and PSKL.DM appear, respectively, as purple and white colonies on this medium, and these were picked and transferred to a minimal salts agar (12) containing either methylene blue (0.0005%), malachite green (25 ng/mL), or azure blue (0.0125 g/L). Isolates producing decolorized zones around colonies on all three dye-containing media were purified by single-colony streaking on Luria agar (13). For DNA extraction, using a PureLink Genomic DNA Mini Kit (Invitrogen) following the manufacturer’s protocol, a single colony was transferred to Luria broth and incubated overnight.

Paired-end short-read sequencing libraries were prepared using the NovaSeqX Series 10B Reagent Kit and sequenced via the Illumina NovaSeq 6000 platform (SP flowcell PE250, v1.5 Reagent Kit) with reads quality-filtered and trimmed using Trimmomatic v0.32 (14). Long-read libraries, prepared from the same extracted DNA using the Rapid Barcoding Kit SQK-RBK004 (without shearing or size selection), were sequenced using a Spot-ON Flow Cell (vR9), MinION sequencer, and MinKNOW v3.1.8 software, with base-calling by Guppy v2.1.3 (all by Oxford Nanopore Technologies plc). The final long-read data set was trimmed by Porechop v0.2.4 (15). Default parameters were used for all software unless otherwise specified.

NovaSeq and MinION data sets were combined using Unicycler v0.4.8-beta (16) to yield single chromosomes of 4,844,288 bp (61.50% G+C) and 4,820,799 bp (61.49% G+C) for PSKL.DP and PSKL.DM respectively, which were submitted to NCBI PGAP v6.5 (17) for annotation. Using GenomesDB (18), JSpeciesWS v.5.0.1 (19) found PSKL.DP and PSKL.DM to share average nucleotide identities (ANIb by BLAST+ [20]) of 96.84% and 97.04% with type strain *Aeromonas hydrophila* subsp. *hydrophila* ATCC7966 (CP000462) and with *Aeromonas dhakensis* 1706-28330 (CP054854) respectively. Sequencing and assembly data are given in Table 1.

**TABLE 1:** Sequencing, assembly, and analysis for PSKL.DP and PSKL.DM, including the loci of putative dye-degrading genes

	PSKL.DP		PSKL.DM	
Sequencing method	Illumina NovaSeq	Nanopore MinION	Illumina NovaSeq	Nanopore MinION
Raw reads	6,317,280	607,929	7,142,248	1,427,792
Mean length bp	250	4,892	250	5,942
Total Gbp	1.58	2.97	1.79	8.48
N50	250	8,648	250	10,900
SRA (raw)	SRX28704585	SRX21625239	SRX28776443	SRX21353834
Filtered reads	6,303,476	110,340	7,131,554	46,964
Mean length	250	13,594	232	31,940
N50	250	13,767	250	31,423
Total Gbp	1.55	1.50	1.65	1.50
*Assembly*				
Species	*Aeromonas hydrophila* subsp. *hydrophila*		*Aeromonas dhakensis*	
GenBank accession	CP117918		CP117919	
Topology	Circular		Circular	
Size (bp)	4,844,288		4,820,799	
G+C %	61.50		61.49	
Coverage	310×		311×	
Rotated to begin	Not rotated		*dnaA* (A0KEC3)	
CDs (coding)^a^	4,260		4,213	
Pseudogenes^a^	23		27	
rRNA (5S-16S-23S)^a^	11-10-10		11-10-10	
tRNA^a^	126		124	
*Analysis* (loci of dye degradation-linked genes)				
Triphenylmethane oxygenase (*tpmD*) (20, 21)	[not found]		PUB92_03280	
FMN-dependent NADH: quinone azoreductase (*azoR*) (22)	PUB83_20410 PUB83_06035		PUB92_10075 PUB92_03145	
Dyp-type peroxidase (23)	PUB83_13030		PUB92_02640	
Laccase (*lacA*)/multicopper oxidase (*cueO*) (24, 25)	PUB83_20335		PUB92_17505	
Tyrosinase (26)	PUB83_11010		PUB92_16400	
Xenobiotic reductase (*xenB*) (27)	[not found]		PUB83_12610	

^*a*^ Found by NCBI PGAP v6.5 (PSKL.DP)/v6.4 (PSKL.DM).

NCBI PGAP and NCBI BLASTp (21) were used to identify proteins encoded in PSKL.DP and PSKL.DM similar to those reported as dye-degrading in strains of *Aeromonas*. Loci of putative homologs*—i.e*. for triphenylmethane oxygenase (*tpmD*) (22, 23), azoreductase (*azoR*) (24), Dyp-type peroxidase (25), laccase (*lacA*) (26, 27), tyrosinase (28), and xenobiotic reductase (*xenB*) (29)—are given Table 1.

## Data Availability

The complete genome sequences and raw sequence data for PSKL.DP and PSKL.DM are available through NCBI under BioProjects PRJNA933919 and PRJNA933921, respectively, with GenBank and SRA accession numbers given in Table 1.
